# Psychosis Relapse Prediction Leveraging Electronic Health Records Data and Natural Language Processing Enrichment Methods

**DOI:** 10.3389/fpsyt.2022.844442

**Published:** 2022-04-05

**Authors:** Dong Yun Lee, Chungsoo Kim, Seongwon Lee, Sang Joon Son, Sun-Mi Cho, Yong Hyuk Cho, Jaegyun Lim, Rae Woong Park

**Affiliations:** ^1^Department of Biomedical Informatics, Ajou University School of Medicine, Suwon, South Korea; ^2^Department of Biomedical Sciences, Ajou University Graduate School of Medicine, Suwon, South Korea; ^3^Department of Psychiatry, Ajou University School of Medicine, Suwon, South Korea; ^4^Department of Laboratory Medicine, Myongji Hospital, Hanyang University College of Medicine, Goyang, South Korea

**Keywords:** natural language processing, psychotic disorder, recurrence, models, statistical, electronic health records

## Abstract

**Background:**

Identifying patients at a high risk of psychosis relapse is crucial for early interventions. A relevant psychiatric clinical context is often recorded in clinical notes; however, the utilization of unstructured data remains limited. This study aimed to develop psychosis-relapse prediction models using various types of clinical notes and structured data.

**Methods:**

Clinical data were extracted from the electronic health records of the Ajou University Medical Center in South Korea. The study population included patients with psychotic disorders, and outcome was psychosis relapse within 1 year. Using only structured data, we developed an initial prediction model, then three natural language processing (NLP)-enriched models using three types of clinical notes (psychological tests, admission notes, and initial nursing assessment) and one complete model. Latent Dirichlet Allocation was used to cluster the clinical context into similar topics. All models applied the least absolute shrinkage and selection operator logistic regression algorithm. We also performed an external validation using another hospital database.

**Results:**

A total of 330 patients were included, and 62 (18.8%) experienced psychosis relapse. Six predictors were used in the initial model and 10 additional topics from Latent Dirichlet Allocation processing were added in the enriched models. The model derived from all notes showed the highest value of the area under the receiver operating characteristic (AUROC = 0.946) in the internal validation, followed by models based on the psychological test notes, admission notes, initial nursing assessments, and structured data only (0.902, 0.855, 0.798, and 0.784, respectively). The external validation was performed using only the initial nursing assessment note, and the AUROC was 0.616.

**Conclusions:**

We developed prediction models for psychosis relapse using the NLP-enrichment method. Models using clinical notes were more effective than models using only structured data, suggesting the importance of unstructured data in psychosis prediction.

## Introduction

The lifetime prevalence of psychotic-spectrum disorders, such as schizophrenia spectrum disorder and mood disorders with psychotic features, is ~3%; moreover, these disorders are accompanied by high levels of morbidity and mortality (1, 2). Psychotic experiences are associated with an increased risk of adverse health outcomes (3). Individuals with psychotic disorders have high relapse rates (4) and ~58% of affected individuals will experience a further episode within 5 years of remission from the initial episode (5).

In practice, early detection and intervention in psychosis have long been considered crucial because they could reduce the severity of relapse or prevent its occurrence (6, 7). Psychosis relapse is associated with poorer occupational and social functioning and more severe symptoms (8, 9). Several studies identified some predictors associated with relapse of psychosis such as poor adherence to treatment (including medication adherence), poor social support, and comorbidities with active psychiatric disorders (10–12). Also, Alvarez-Jimenez et al. (13) mentioned that structured clinical variables and general demographics might have a lower impact on the relapse rates than adherence or social functioning in their meta-analysis. However, it is difficult to utilize these predictors because they are not usually collected directly, and the recorded data format is heterogeneous (14).

A transdiagnostic approach using clinical predictors had been previously attempted to overcome this issue and exhibited a high accuracy (15). Various studies examined the application of natural language processing (NLP) to mental disorders even though privacy concerns limited the accessibility to data sources. Adequately expressing mental illness with only structured data is difficult, and the information on important psychiatric clinical context is usually recorded as free text in clinical notes (16); utilizing such unstructured data might be crucial in psychiatry. Therefore, various NLP techniques could be useful in detecting or identifying patients at risk of various psychiatric disorders. Researchers could use sentimental analysis to predict depression (17), document classification methods to predict suicide attempts (18), semantic relationships to predict anxiety (19), and NLP-derived predictors to predict psychosis (20). However, studies predicting psychosis relapse using the NLP technique are rare, despite these potential advantages, and have not been validated.

Various types of notes are used for prediction using NLP (21). Among them, the clinical rating scale is useful to classify psychiatric cases (22, 23). The admission notes are frequently used as a reference document for the patient's history in clinical settings (24), and nursing notes also record important signals about the patient's condition and clinical outcome. These records help predict outcomes and identify risk factors (25). Although predictive models using each note type or multiple notes simultaneously are being developed, the most valuable notes for NLP have not yet been established.

In this study, we aimed to develop prediction models for psychosis relapse with the NLP technique using various types of clinical notes in addition to structured data. We also compared the performance of models using both structured data and clinical notes and a model based on structured data only and externally validated the performance of both.

## Methods

### Data Source

The clinical data for the model derivation were extracted from the electronic health records (EHRs) of the Department of Psychiatry and Mental Health Center at the Ajou University Medical Center (AUMC) in South Korea between 2012 and 2020 (26). All patients in the database had at least one psychiatric diagnosis (ICD-10-CM codes F00-F99). Only patients diagnosed by psychiatrists and psychologists using assessment scales were selected from the database to clearly classify patients with mental illness, and a total of 1,986 patients were collected. A model was developed by extracting the data of patients who met the criteria from the records of 1,986 patients. The clinical data included socio-demographics, diagnoses, medications, procedures, laboratory tests, and clinical notes. In particular, the admission notes, initial nursing assessment notes, and psychological test notes were extracted. The documents were limited to the nearest record within 1 month before the index date. And the source texts of the document were 33% in English and 67% in Korean, similar by patient, document, and database. We used the AUMC database formatted according to the Observational Medical Outcomes Partnership–Common Data Model (OMOP-CDM) version 5.3.1, maintained by the Observational Health Data Sciences and Informatics (OHDSI) and de-identified (27).

Furthermore, another EHR database was used for the external validation of the predictive models developed. The Myongji Hospital (MJH) database in South Korea has data from 882,646 patients who visited hospital from 2003 to 2021. The MJH database was also included in the OMOP-CDM version 5.3.1. In contrast to the AUMC database, there was no separate classification process for psychiatric cases in the Myongji Hospital. Data of the same types as those in the AUMC database were extracted.

This study was approved by the Institutional Review Board of the Ajou University Hospital (AJIRB-MED-MDB-21-151), and informed consent was not required due to the use of de-identified data. Access to the MJH database during the external validation process was allowed under the IRB mutual recognition agreement (Research Free Zone agreement).

### Clinical Notes

#### Psychological Tests

These notes contained clinical and cognitive function assessments conducted by psychiatrists and clinical psychologists. It also included the developmental history, brief past history, and diagnosis. The assessments included the Korean version of the Wechsler Adult Intelligence Scale-III (WAIS) for intelligence quotient (IQ), the Trail Making Test for processing speed, the Wisconsin Card Sorting Test (WCST) for perseverative errors, the Korean version of the verbal fluency task for semantic fluency, the Korean version of the California Verbal Learning Test (K-CVLT) for verbal memory, the Beck Depression Inventory (BDI) for depression, the State-Trait Anxiety Inventory (STAI) for anxiety, and the Minnesota Multiphasic Personality Inventory-2 (MMPI-2). The psychological test notes did not include the scores of each test, only the parts described in the psychologist's interpretation and the score evaluations, to increase comparability by note. For example, when the patient's IQ was ≤ 70, the only description included was “the patient's overall intelligence was below average.”

#### Admission Notes

These notes included various types of information, personal (age, sex, family composition, location, level of education, and personality), medical history (past medical history, family history, social history, past psychiatric history, and history of present illness and symptoms), and the psychiatric assessment (diagnosis, medication, and plan). These notes were recorded by a psychiatrist.

#### Initial Nursing Assessment

These notes mainly recorded social information, current status, and the transfer pathway. Social information comprised religion, alcohol intake, smoking, and interpersonal relationships; current status included prominent symptoms and symptoms that required attention, such as aggression or violence. The transfer pathway referred to information about the patient's path, such as whether they were hospitalized from an outpatient clinic, another hospital, or another ward. These notes were recorded by a psychiatric ward nurse.

### Study Population and Outcome

The study population included patients with psychotic disorders, including schizophrenia spectrum disorders, mood disorders with psychotic symptoms, and other psychotic disorders, and health records covering more than 365 days. The index date was defined as the patient's first record of a diagnosis of psychotic disorder. Exclusion criteria were the absence of antipsychotic prescriptions and psychiatric procedures after the index date.

The definition of study outcome was relapse of psychosis within 1 year after the index date. Relapse was defined as emergency department visits or hospitalization due to exacerbation of a patient with psychosis. In addition, re-hospitalization after discharge for the first diagnosis was included (28). We binarized the outcomes into relapse and non-relapse based on the occurrences recorded only in the main database. A detailed code list of the clinical diagnoses, medications, procedures, laboratory tests, and visit concepts in this study is provided in the online Supplementary Material.

### Model Development

**Figure 1:**
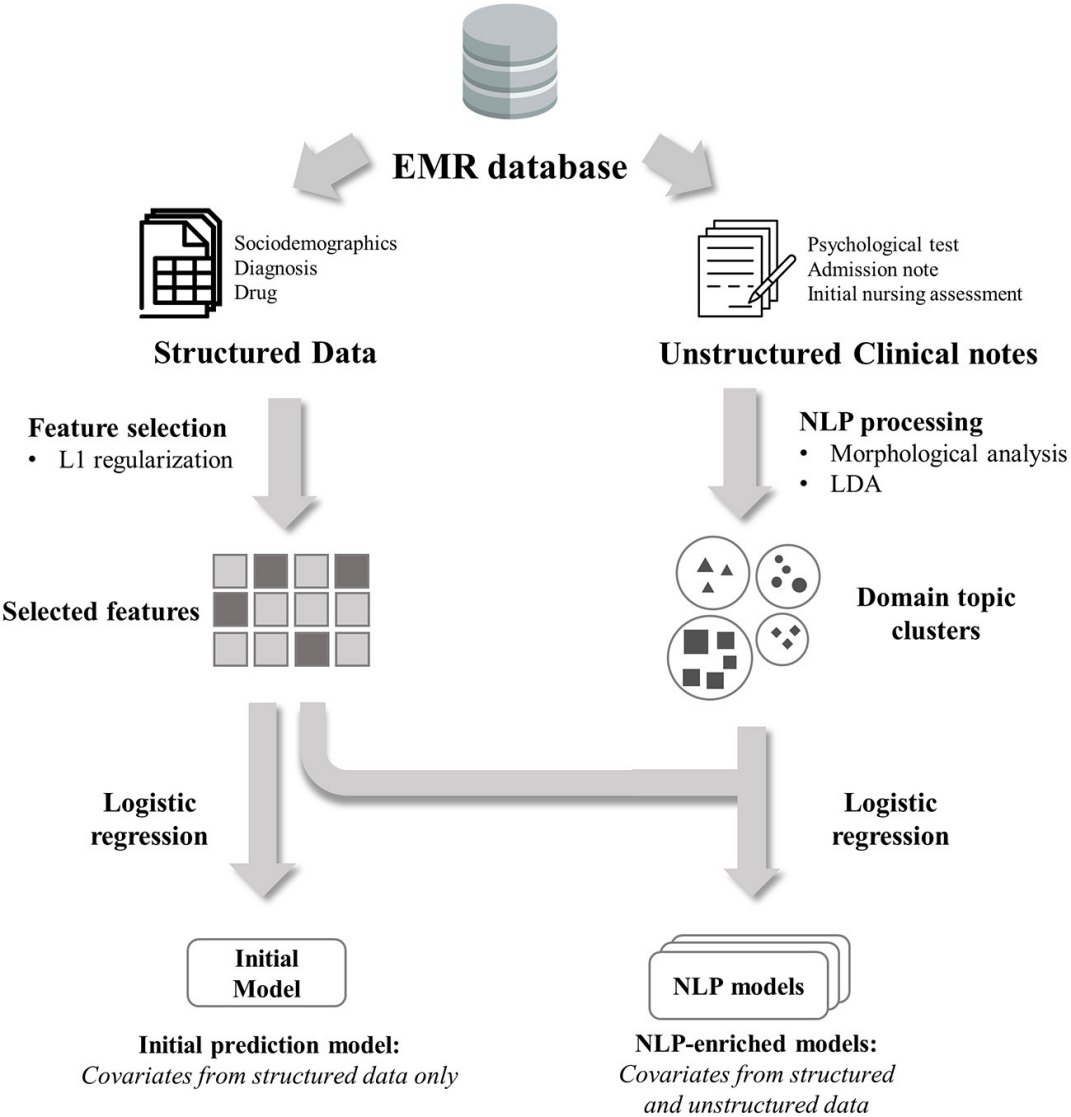
Overview of model development process. Initial model was developed using selected features only from structured data. NLP-enriched models were developed using selected features plus features from unstructured clinical notes.

We developed a model based on structured data alone (initial model) and other models with both structured data and unstructured free text (NLP-enriched models) (Figure 1). These models were separately developed according to the clinical notes used, and their performances compared. We used the patient-level prediction (PLP) framework of the OHDSI to develop and validate these predictive models. This framework consisted of standardized model development and validation processes that require defining predictable problems and selecting the study population, outcome, population settings, predictors, and statistical algorithms (29).

#### Initial Model (Model 1)

The predictive variables for model training were extracted and dichotomized for existence within short-term (-30 days) and long-term (-365 days) intervals prior to the index date to capture the medical history temporality of the psychiatric cases. The variables included patient demographics (sex and age in 5-year groups), condition group (medical diagnosis, grouped using a SNOMED–CT hierarchy), drug group (based on the active ingredients), procedure (e.g., psychotherapy, electroconvulsive therapy.), measurement (e.g., assessment scale, laboratory test.) and observation (e.g., smoking status, alcohol intake). Predictors not recorded in our EMR system were considered non-occurring. Through this process, 6,069 candidate variables were generated. For feature selection, we conducted the least absolute shrinkage and selection operator method (LASSO) and selected the final predictors with variable importance and clinical relevance. The initial prediction model was developed using logistic regression with selected variables.

#### NLP-Enriched Models (Models 2, 3, 4, and 5)

We developed the NLP-enriched models using the final variables from the initial model and additional NLP-derived variables. Three models were developed, one for each type of clinical notes (admission note, initial nursing assessment note, and psychological test note), and one model using all types of clinical notes. NLP algorithms were used to extract the topics as predictive variables from each clinical document. In pre-processing, we performed a morphological analysis that automatically indexes morphological forms in the documents rather than the vocabulary itself (30). We filtered nouns in the Korean text and the entire text for English documents. Then, we converted the documents into a bag-of-words model of the corpus after pre-processing, including stemming, normalization, and stop word removal. Latent Dirichlet allocation (LDA), an unsupervised learning method, was used to cluster the topics from each document (31). With an LDA-based topic model, the topic probabilities were calculated for each note. For instance, if 10 topics were created by the LDA from the admission notes, the probability of being assigned to 10 topics for each admission note was generated. We developed models with the addition of covariates that were probability values obtained using the NLP algorithm. Before using the LDA, we also calculated the perplexity scores to determine the optimal number of topics in the LDA (32). Using the perplexity score can estimate relative quality of statistical models (33). The study population was randomly split into the training set (75%) and the test set (25%) to develop the initial and enriched models, and 3-fold cross-validation was conducted within the training data set.

### External Validation

We conducted an external validation to confirm the validity of the model performance using a different dataset from the MJH database. All settings and evaluation processes were conducted using the same methods as in the model development. However, distinguishing the MJH database admission notes from the psychiatry department was not possible due to their formatting difference from the clinical notes in the AUMC database; furthermore, the psychological test results were not available. Therefore, only two models were validated–the initial model (model 1) and the enriched model developed using the initial nursing assessment notes (model 4), tagged as being from the Department of Psychiatry.

### Statistical Analysis

All the variables were appropriately summarized. The baseline demographic and clinical characteristics (medical history, psychiatric history, and psychiatric medication use) are presented as counts (percentage) for categorical variables, and mean and standard deviation or median and interquartile range for continuous variables. The chi-square test was used to compare categorical variables between populations. In all analyses, *p*-values < 0.05 were considered statistically significant. For the predictive model evaluation, we calculated four metrics: accuracy, F1 score, area under the precision and recall curve (AUPRC), and area under the receiver operating characteristic curve (AUROC). We used the maximal Youden index to select the optimal cut-off value in each prediction model and calculated the accuracy using its cut-off value (34).

All analyses were performed using R software version 3.6 (R Foundation for Statistical Computing, Vienna, Austria), OHDSI's Health Analytics Data to Evidence Suite (HADES) packages, and open-source statistical R packages. All source codes are available in the GitHub repository (https://github.com/ABMI/PsychosisMultimodalValidation).

## Results

### Baseline Characteristics

Table 1 shows the baseline characteristics of the study population. A total of 330 patients were selected according to the inclusion and exclusion criteria. When the patients were grouped by age at 10-year intervals, we noted that patients in their 20's had a high incidence of relapse (39.2% and 53.2% of patients were in their 20's in the non-relapse and relapse groups, respectively; *p* = 0.047). There was no significant difference in sex and medical history between the groups. Moreover, the proportion of schizoaffective disorder in the relapse group was 0%, significantly lower than the 9.7% in the non-relapse group (*p* < 0.01). The proportion of mood and anxiety disorders was also significantly lower in the relapse group (*p* < 0.01 and *p* = 0.04, respectively). In terms of psychiatric medication use, antipsychotics and antidepressants were significantly less used in the relapse group (*p* < 0.01 and *p* < 0.01, respectively).

**Table 1:** Baseline characteristics for study population with or without relapse.

**Variable**	**Non-relapse (*n* = 268)**	**Relapse (*n* = 62)**	** $X_{\left(df\right)}^{2}$ **	***P*-value**
**Age group**, ***n*** **(%)**
<20	63 (23.5)	8 (12.9)	3.35 (1)	0.07
20–29	105 (39.2)	33 (53.2)	4.08 (1)	0.04^*^
30–39	60 (22.4)	11 (17.7)	0.64 (1)	0.42
v≥40	40 (14.9)	10 (16.1)	0.06 (1)	0.81
**Sex**, ***n*** **(%)**
Male	115 (42.9)	27 (43.5)	0.01 (1)	0.93
**Medical history**, ***n*** **(%)**
Diabetes mellitus	1 (0.3)	0 (0.0)	0.23 (1)	0.63
Heart disease	2 (0.7)	0 (0.0)	0.47 (1)	0.50
Hypertension	11 (4.1)	1 (1.6)	0.89 (1)	0.34
**Psychiatric history**, ***n*** **(%)**
Acute transient psychotic disorder	45 (16.8)	12 (19.4)	0.23 (1)	0.63
Anxiety disorder	21 (7.8)	1 (1.6)	3.13 (1)	0.08
Delusional disorder	14 (5.2)	2 (3.2)	0.44 (1)	0.51
Insomnia	13 (4.9)	1 (1.6)	1.30 (1)	0.25
Mood disorder	103 (38.4)	3 (4.8)	26.06 (1)	<0.01^*^
Neurodevelopmental disorder	12 (4.5)	2 (3.2)	0.19 (1)	0.66
Schizoaffective disorder	26 (9.7)	0 (0.0)	6.53 (1)	0.01^*^
Schizophrenia	129 (48.1)	38 (61.3)	3.49 (1)	0.06
**Psychiatry medication use**, ***n*** **(%)**
Anticholinergics	19 (7.1)	1 (1.6)	2.65 (1)	0.10
Antidepressants	214 (80.0)	22 (35.5)	48.65 (1)	<0.01^*^
Antiepileptics	12 (4.5)	2 (3.2)	0.19 (1)	0.66
Antipsychotics	219 (81.7)	17 (27.4)	62.98 (1)	<0.01^*^
Benzodiazepine	156 (58.2)	17 (27.4)	19.14 (1)	<0.01^*^
Beta blocking agents	19 (7.1)	2 (3.2)	1.26 (1)	0.26
Opioids	11 (4.1)	0 (0.0)	2.63 (1)	0.10

*$\chi_{\left(df\right)}^{2}$, chi square value and degree of freedom*;

* *Indicates statistical significance (P-value <0.05)*.

### Model Specification

As part of the variable selection, six predictors were selected through L1 regularization among a total of 6,069 candidate predictors and were used in the initial and NLP-enriched models. The characteristics selected were male sex, non-drinker, non-smoker, and exposure to antipsychotic drugs, individual psychotherapy, and diagnosis of depression within a year before the index date.

We selected 10 topics as the most reliable hyperparameters for LDA performance (Online Supplementary Figure 1) for each NLP-enriched model based on the perplexity scores. Each topic had the probability of being assigned to the topic as the variable value. These topics were added to the six predictors selected in the initial model. Each model was implemented by selecting only some of the topics in the final prediction process. Table 2 lists the topics finally used for each model. Five topics were selected from the psychological tests; three related to intellectual function (i.e., borderline disability, developmental, and normal), and two to mood symptoms (anxiety and bipolar I disorder). Only one topic was selected and included in the model from the admission notes, comprising delusion, persecutory disorder, and irritability. Three topics were selected from the initial nursing assessment notes; one related to alcohol intake, aggressive behavior, and psychosis; the second included depression and bipolar I disorder, and the last was related to marginal, withdrawal, and self-talk.

**Table 2:** Domains selected by LASSO model in admission note, a note of psychological tests, and a note of initial nursing assessment.

**Note type**	**Main features of the domain**	**Topic examples of the domain**
Psychological test	Intellectual function, borderline disability, moderate disability, poor	Psychotic, intellectual, disability, 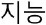 (intellectual function), intellectual functioning, moderate disability, borderline disability, borderline intellectual disability, 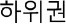 (low level), poor
	Developmental, early onset, functioning	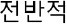 (overall), 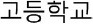 (high school), 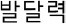 (developmental history), functioning, 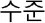 (level), 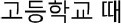 (in high school), 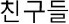 (friends)
	Normal level, possibility, potential intelligence	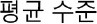 (normal level), 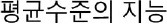 (normal level of intellectual function), 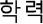 (academic background), 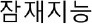 (potential intelligence), possibility
	Mood, schizophreniform, anxiety, agitation	stress, anxiety, schizophreniform, auditory hallucination, mood, delusional, psychomotor agitation, panic attack, non-functioning, anger, personality, disorder
	Bipolar I disorder, manic episode, psychotic features	manic, manic episode, bipolar I disorder, current episode, mood-congruent psychotic features, depressive, severe, self-talking, 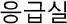 (emergency room), acute episode, manic severe, aggressive behavior, sleep disturbance
Admission notes	Delusion, persecutory, disorder, irritable, parent history	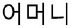 (mother), 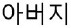 (father), disorder, persecutory, personal, delusional, medication, 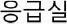 (emergency room), acute, persecutory delusion, delusional disorder, irritable, auditory, talkative, panic, seroquel, 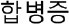 (complication), 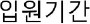 (length of stay), problem, onset, illness
Initial nursing assessment	Admission, alcohol intake, first, aggressive, psychotic	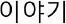 (story), 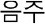 (alcohol intake), marginal, 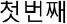 (first), 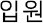 (admission), 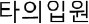 (involuntary admission), 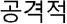 (aggressive), 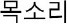 (auditory), persecutory delusion, 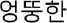 (inappropriate), 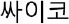 (psychotic), anxiety, 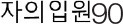 90(voluntary admission)
	Depression, bipolar I disorder, persistent	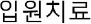 (inpatient treatment), stress, 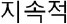 (persistent), 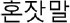 (self-talking), 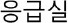 (emergency room), 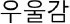 (depression), 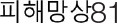 81(persecutory delusion), Dystonia, marginal, major depressive disorder, bipolar I disorder
	Adjustment, marginal, withdrawal, self-talking, resistant	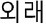 (outpatient), delusion, disturbance, adjustment disorder, Idea of reference, withdrawal, adjustment, marginal, withdrawal, ativan, diazepam, self-talking, stress, 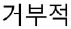 (resistant)

### Model Performance

**Figure 2:**
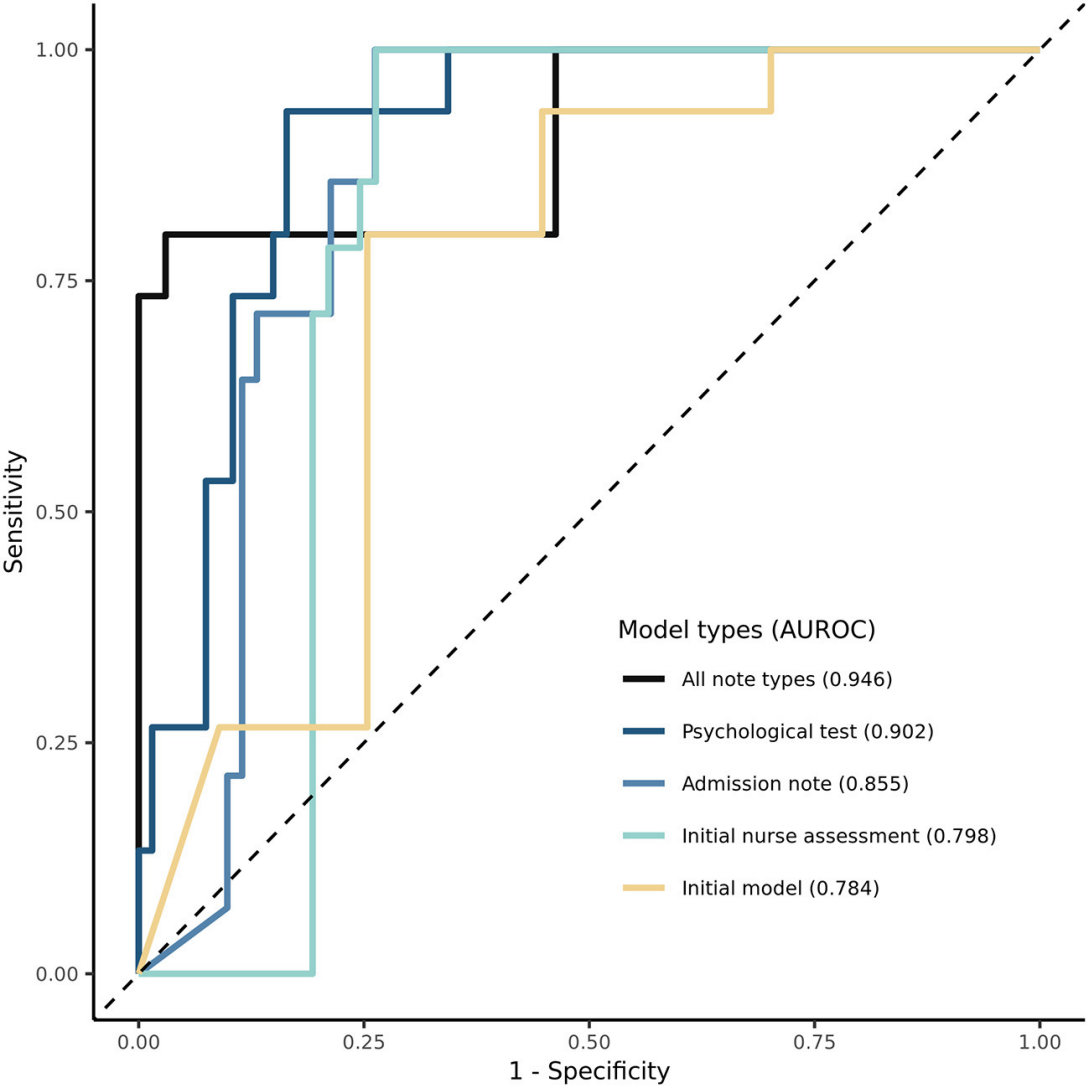
Receiver Operating Characteristic (ROC) curve of models predicting relapse in psychosis. The ROC curve for initial model and NLP-enriched models is shown. Performance of models using area under the receiver operating characteristic curve (AUC) is compared.

Among the 330 patients with psychotic disorders in the AUMC database, 62 (18.8%) experienced a psychosis relapse. The mean interval to relapse was 35 days. The initial model had an accuracy of 0.775, an F1 score of 0.595, an AUPRC of 0.362, and an AUROC of 0.784 in the internal cross-validation dataset (Table 3). Figure 2 shows the ROC curves of the initial model and the NLP-enriched models, obtained using logistic regression. In terms of accuracy, F1 score, and AUROC, all the enriched models with unstructured covariates had a higher performance than the initial model in the internal validation dataset (all note types: 0.900, 0.705, 0.946; psychological tests: 0.842, 0.675, 0.902; admission notes: 0.835, 0.686, 0.855; and initial nursing assessment: 0.835, 0.697, 0.798, respectively). Among the enriched models, the one using all note types had the highest performance. Among the enriched models for each type of clinical notes, the one based on the psychological tests had the highest accuracy, AUPRC, and AUROC (0.842, 0.625, and 0.902, respectively, internal validation). The calculated importance of the variables in each model is shown in Supplementary Table 1.

**Table 3:** Performance results of the initial model and NLP-enriched models using the clinical note.

**Performance metrics**	**Initial model (Model 1)**	**Psychological test (Model 2)**	**Admission notes (Model 3)**	**Initial nursing assessment (Model 4)**	**All note types (Model 5)**
ACC	0.775	0.842	0.835	0.835	0.900
AUPRC	0.362	0.625	0.407	0.340	0.883
AUROC	0.784	0.902	0.855	0.798	0.946
F1 score	0.595	0.675	0.686	0.697	0.705

*ACC, accuracy; AUPRC, area under the precision recall curve; AUROC, area under the receiver operating characteristics curve*.

### External Model Validation

The external validation of the initial model and enriched model 4 was conducted using the MJH database. Among the 4,391 patients identified in the external validation dataset, 202 (4.6%) experienced psychosis relapses. The mean interval to relapse was 80 days. The external validation performance of the initial model had an accuracy of 0.114, an F1 score of 0.089, an AUPRC of 0.042, and an AUROC of 0.468. In contrast, the NLP-enriched model using the initial nursing assessment had an accuracy of 0.832, an F1 score of 0.209, an AUPRC of 0.097, and an AUROC of 0.616 (Online Supplementary Table 2).

## Discussion

In this study, we developed prediction models for psychosis relapse using NLP enrichment methods; these models demonstrated higher performance than the initial model using only structured data, as the traditional approach. Furthermore, we compared the models according to the type of clinical notes used and found that the model based on psychological tests provided the highest performance compared to the other enriched models for each type of clinical notes. External validation was performed using a different database converted into the same data type, and showed that the enriched model was more effective than the structured-data model. It has been recently reported that models with NLP features have higher predictive performance (20), and this result was confirmed in our study. However, how the performance differs depending on the type of clinical notes used remains unclear, and the present study investigated which note types are more helpful to predictive performance.

We extracted the characteristic information of each note type using the LDA method. LDA can reportedly reflect and represent the semantic characteristics of the document through topic clustering of similar words (35). Moreover, LDA can treat all notes as if written in on common language, despite originally being in a mix of Korean and English in our data (36). Furthermore, the LDA method is relatively straightforward to understand since it reflects semantic characteristics compared to other machine learning techniques using black-box algorithms and insufficient transparency (37). Thanks to these advantages, prediction models using the LDA method have been used in several studies (38, 39).

We compared the performance of the prediction models based on the type of notes used. Previous studies on prediction models used admission notes, discharge notes, nursing notes, and notes with psychological scales (38, 40, 41). In addition, prediction models have been created through the indiscriminate use of several notes (42, 43). However, to the best of our knowledge, no study has compared prediction models based on the type of notes used. Although the model that used all note types showed the most reliable performance, limited data is commonly used for clinical prediction (44); therefore, it may be useful to determine which data type is more helpful for this purpose.

Among more than 6,000 candidate predictors from the structured data, we identified six predictors for the model development, consistent with the results of previous studies. In a systematic review of prediction models for psychosis relapse (45), a history of prior health services and symptoms of depression were used in the prediction model. Other studies have found that substance use, including alcohol consumption and smoking, was significantly associated with relapse (13, 46). Moreover, the relapse rate in relation to transfer is also reportedly greater for females than for males (47).

Since admission notes usually contain information on past history, family history, and current status (48), we extracted parent history and current status such as delusion and irritability from these notes. Similarly, from the psychological tests, we extracted the test-related developmental history, intelligence, and symptoms of depression and anxiety, and from the initial nursing assessment, the alcohol intake, aggressiveness, involuntary admission associated with the patient's condition, social information, and transfer pathway (49). After comparing the performance of the enriched models based on each type of clinical note, the model using the psychological tests emerged as the most promising. This result is consistent with previous studies reported high-performance predictive models using clinical assessment or developmental history, such as psychological tests (50, 51). Specifically, a lower IQ reportedly predicts worse outcomes in psychosis (52). In the model using the psychological tests, topics related to low IQ were used as variables that predict an increased likelihood of relapse. In addition, through symptoms measured by the BDI and STAI, topics related to severe anxiety and depression were also used as variables, including low IQ. Both depression and anxiety are associated with the severity of psychosis (53). Furthermore, early onset is reportedly associated with negative outcomes (54) and was also used as a variable in our models. However, it should be noted that the variable of early onset was used as a variable to predict a reduced chance of relapse, since most of the patients with such records in our study were diagnosed early. Ultimately, early detection improves the outcomes of psychotic disorders (55). Thus, our findings suggest that psychological tests are a useful note type for predicting psychosis relapse.

Interestingly, despite using already validated predictors in the initial model, the performance of the external validation was poor, and the predictability was lost. The external validation database appears to have lower performance because it does not distinguish psychiatric patients in advance. The data of 4,391 patients have been extracted from the Myongji Hospital, a markedly larger sample than from the AUMC database (330 patients); however, the Myongji Hospital had mixed cases, including non-psychiatric. In fact, it is difficult to extract psychiatric symptoms or records from data (56). For this reason, some psychiatric cases are registered separately from the EHR data for psychiatric research (57). In addition to the classification problem for these psychiatric cases, general difficulties in the external validation of prediction models have been previously reported. Other studies have shown that prediction models exhibited poor performance during external validation for some prediction tasks (58, 59). Due to poor reproducibility and generalizability, the implementation of prediction models in clinical settings is limited despite the development of various models. More diverse data sources could be the solution to this problem, and it has recently been reported that adding free text to data sources using NLP enhances the predictive power (20, 37, 60). In this regard, our study developed models using NLP features, confirming an improved performance compared to the initial model based only on structured data. Moreover, the performance of the NLP-based model in the external validation was higher than that of the initial model. Although the improved model performance during external validation was insufficient, we enhanced the result using only the initial nursing assessment, the least effective among the NLP models.

This study has several limitations. First, we could not include external data from different hospitals due to the limited hospital data. More complete claim data must be used to overcome this limitation. However, claim data does not include unstructured data such as admission note; therefore, our research design could not be performed as such. In this regard, as with other studies using EMR, those not recorded were considered to have not occurred. Second, according to the definition of relapse, relapse cases with an outpatient visit or visits to other hospitals were not included in this study. Defining relapse with an outpatient diagnosis and prescription has a practical limitation. Therefore, we tried to limit conditions with hospitalization or emergency room visit. Inpatient records from other hospitals could not be obtained. This can be overcome by linking with other hospital data and with health insurance claim data, but there are practical difficulties. Third, when defining relapse, we did not distinguish causes such as manic episode and drug-induced psychosis. However, individuals with psychotic symptoms in actual clinical practice have comorbid symptoms, such as substance misuse and anxiety (61). Fourth, external validation of the model was performed using only the data from one hospital, and performance comparison by note was not performed. Psychiatric records are often managed separately or are not disclosed (62), making it difficult to find hospital records that could be used for external validation; furthermore, it is more difficult to distinguish various types of records. Future research is therefore needed to investigate and validate our results. Fifth, the Myongji Hospital database used in external validation had different characteristics from Ajou University database. Unlike 18% of patients in the Ajou University database, 4.6% of patients experienced a relapse in the Myongji Hospital database. In the population of MJH database, the proportion of patients in their 40's or older was higher than that of the developing dataset. Also, unlike the developing dataset, there were significant differences between the groups with and without outcomes in medical history (diabetes mellitus and hypertension), psychiatric history (insomnia and neurodevelopmental disorder), and psychiatry medication (anticholinergics). These differences seem to have affected the difference in relapse rates (Online Supplementary Table 3). And there was a difference in the mean duration to relapse. In both the Ajou University Medical Center and the Myongji hospital, most of the patients relapsed within the first 3 months. Although the median was shorter at the AUMC than at the MJH, this can be explained by the fact that 6% of MJH's relapse was emergency room visits and AUMC's 22% were emergency room visits (Online Supplementary Table 5). It has been reported that there were differences according to institutions for psychiatric patients in South Korea (63). In addition, it is rather desirable to use data with different features to evaluate the generalizability of the model (64). Sixth, when defining patients with psychotic disorder, we included both schizophrenia spectrum and affective psychoses. Because of that, we could not use some specific criteria such as PANSS. However, there is diagnostic uncertainty of first episode psychosis, especially in electronic health data (65). In addition, the highest diagnostic conversion rate from unipolar depression to schizophrenia emerged during the first year (66). Actually, other studies have included both schizophrenia spectrum and affective psychoses in psychotic disorders (67, 68). In this regard, we tried to develop the general model for early relapse prediction of first episode psychosis. Seventh, due to the limitation of the observational database, we could not include the length of the disorder and the duration of the untreated disorder as covariates. Eighth, considering the topic correlation, we determined the appropriate topic number with CaoJuan2009 metric in the LDA model. Although the model was developed in consideration of the topic correlation, overlapping topics makes clinical interpretations unclear. Ninth, in Table 1, there was no statistically significant difference between two groups for acute transient psychotic disorder which had a tendency to relapse. It appears to be due to a problem with the limited number of patients, and further studies are needed in the future.

In summary, we utilized three types of clinical notes to predict clinical relapse in patients with psychotic disorders. Clinical relapse could be more effective predicted using NLP-based models than a model based only on structured data. Furthermore, we found that the predictive model based on the psychological tests provided the highest predictive performance. In clinical situations with large data heterogeneity for each patient, our findings suggest that which type of note would be more useful to use.

## Data Availability Statement

The original contributions presented in the study are included in the article/Supplementary Materials, further inquiries can be directed to the corresponding author.

## Ethics Statement

The studies involving human participants were reviewed and approved by the Institutional Review Board of the Ajou University Hospital (AJIRB-MED-MDB-21-151). Written informed consent for participation was not required for this study in accordance with the national legislation and the institutional requirements.

## Author Contributions

DL and CK contributed to data analysis, and writing the manuscript. S-MC, YC, JL, and SS gave critical opinions on the study design, and the manuscript. SL and RP contributed to interpreting the results, and supervised the entire process. All authors contributed to the literature review, study design, data interpretation, and approved the final manuscript.

## Funding

This research was funded by the Bio Industrial Strategic Technology Development Program (20003883 and 20005021), the Ministry of Trade, Industry and Energy (MOTIE, Korea) and a grant from the Korea Health Technology R&D Project through the Korea Health Industry Development Institute (KHIDI), the Ministry of Health and Welfare, Republic of Korea (Grant Number: HR16C0001), and Basic Science Research Program through the National Research Foundation of Korea (NRF) funded by the Ministry of Education [NRF-2020R1I1A1A01072208].

## Conflict of Interest

The authors declare that the research was conducted in the absence of any commercial or financial relationships that could be construed as a potential conflict of interest.

## Publisher's Note

All claims expressed in this article are solely those of the authors and do not necessarily represent those of their affiliated organizations, or those of the publisher, the editors and the reviewers. Any product that may be evaluated in this article, or claim that may be made by its manufacturer, is not guaranteed or endorsed by the publisher.

## References

[1] Perälä J Suvisaari J Saarni SI Kuoppasalmi K Isometsä E Pirkola S. Lifetime prevalence of psychotic and bipolar I disorders in a general population. Arch Gen Psychiatry. (2007) 64:19–28. 10.1001/archpsyc.64.1.19 17199051

[2] Breitborde NJ Moe AM Ered A Ellman LM Bell EK. Optimizing psychosocial interventions in first-episode psychosis: current perspectives and future directions. Psychol Res Behav Manag. (2017). 10.2147/PRBM.S111593 28490910 PMC5414722

[3] Oh H Koyanagi A Kelleher I DeVylder J. Psychotic experiences and disability: findings from the collaborative psychiatric epidemiology surveys. Schizophr Res. (2018) 193:343–7. 10.1016/j.schres.2017.07.049 28797526 PMC5912340

[4] Martland N Martland R Cullen AE Bhattacharyya S. Are adult stressful life events associated with psychotic relapse? a systematic review of 23 studies. Psychol Med. (2020) 50:2302–16. 10.1017/S0033291720003554 33054892

[5] Lally J Ajnakina O Stubbs B Cullinane M Murphy KC Gaughran F. Remission and recovery from first-episode psychosis in adults: systematic review and meta-analysis of long-term outcome studies. Br J Psychiatry. (2017) 211:350–8. 10.1192/bjp.bp.117.201475 28982659

[6] McGorry PD Killackey E Yung A. Early intervention in psychosis: concepts, evidence and future directions. World psychiatry. (2008) 7:148. 10.1002/j.2051-5545.2008.tb00182.x 18836582 PMC2559918

[7] Eisner E Drake R Barrowclough C. Assessing early signs of relapse in psychosis: review and future directions. Clin Psychol Rev. (2013) 33:637–53. 10.1016/j.cpr.2013.04.001 23628908

[8] Wiersma D Nienhuis FJ Slooff CJ Giel R. Natural course of schizophrenic disorders: a 15-year followup of a Dutch incidence cohort. Schizophr Bull. (1998) 24:75–85. 10.1093/oxfordjournals.schbul.a033315 9502547

[9] Mattsson M Topor A Cullberg J Forsell Y. Association between financial strain, social network and five-year recovery from first episode psychosis. Soc Psychiatry Psychiatr Epidemiol. (2008) 43:947–52. 10.1007/s00127-008-0392-3 18604620

[10] Fikreyesus M Soboka M Feyissa GT. Psychotic relapse and associated factors among patients attending health services in Southwest Ethiopia: a cross-sectional study. BMC Psychiatry. (2016) 16:354 10.1186/s12888-016-1076-2 27765033 PMC5072324

[11] Mi W-F Chen X-M Fan T-T Tabarak S Xiao J-B Cao Y-Z. Identifying modifiable risk factors for relapse in patients with schizophrenia in China. Front Psychiatry. (2020) 11:574763. 10.3389/fpsyt.2020.574763 33061925 PMC7518216

[12] Ahmad I Khalily MT Hallahan B Shah I. Factors associated with psychotic relapse in patients with schizophrenia in a Pakistani cohort. Int J Mental Health Nurs. (2017) 26:384–90. 10.1111/inm.12260 27704675

[13] Alvarez-Jimenez M Priede A Hetrick SE Bendall S Killackey E Parker AG. Risk factors for relapse following treatment for first episode psychosis: a systematic review and meta-analysis of longitudinal studies. Schizophr Res. (2012) 139:116–28. 10.1016/j.schres.2012.05.007 22658527

[14] Adler DA Ben-Zeev D Tseng VW Kane JM Brian R Campbell AT. Predicting early warning signs of psychotic relapse from passive sensing data: an approach using encoder-decoder neural networks. JMIR mHealth and uHealth. (2020) 8:e19962. 10.2196/19962 32865506 PMC7490673

[15] Fusar-Poli P Werbeloff N Rutigliano G Oliver D Davies C Stahl D. Transdiagnostic risk calculator for the automatic detection of individuals at risk and the prediction of psychosis: second replication in an independent national health service trust. Schizophr Bull. (2019) 45:562–70. 10.1093/schbul/sby070 29897527 PMC6483570

[16] Jackson RG Patel R Jayatilleke N Kolliakou A Ball M Gorrell G. Natural language processing to extract symptoms of severe mental illness from clinical text: the clinical record interactive search comprehensive data extraction (CRIS-CODE) project. BMJ Open. (2017) 7:e012012. bmjopen-2016-012012 10.1136/bmjopen-2016-012012 28096249 PMC5253558

[17] Babu NV Kanaga EGM. Sentiment analysis in social media data for depression detection using artificial intelligence: a review. SN Comput Sci. (2022) 3:74. 10.1007/s42979-021-00958-1 34816124 PMC8603338

[18] Fernandes AC Dutta R Velupillai S Sanyal J Stewart R Chandran D. Identifying suicide ideation and suicidal attempts in a psychiatric clinical research database using natural language processing. Sci Rep. (2018) 8:7426. 10.1038/s41598-018-25773-2 29743531 PMC5943451

[19] Osadchiy V Mills JN Eleswarapu SV. Understanding patient anxieties in the social media era: qualitative analysis and natural language processing of an online male infertility community. J Med Internet Res. (2020) 22:e16728. 10.2196/16728 32154785 PMC7093775

[20] Irving J Patel R Oliver D Colling C Pritchard M Broadbent M. Using natural language processing on electronic health records to enhance detection and prediction of psychosis risk. Schizophr Bull. (2021) 47:405–14. 10.1093/schbul/sbaa126 33025017 PMC7965059

[21] Kulshrestha S Dligach D Joyce C Baker MS Gonzalez R O'Rourke AP. Prediction of severe chest injury using natural language processing from the electronic health record. Injury. (2021) 52:205–12. 10.1016/j.injury.2020.10.094 33131794 PMC7856032

[22] Núñez D Arias V Méndez-Bustos P Fresno A. Is a brief self-report version of the Columbia severity scale useful for screening suicidal ideation in Chilean adolescents? Compr Psychiatry. (2019) 88:39–48. 10.1016/j.comppsych.2018.11.002 30471550

[23] Zimmerman M Chelminski I McGlinchey JB Posternak MA. A clinically useful depression outcome scale. Compr Psychiatry. (2008) 49:131–40. 10.1016/j.comppsych.2007.10.006 18243884

[24] Ehsanullah J Ahmad U Solanki K Healy J Kadoglou N. The surgical admissions proforma: Does it make a difference? Ann Med Surg. (2015) 4:53–7. 10.1016/j.amsu.2015.01.004 25750727 PMC4348450

[25] Korach ZT Yang J Rossetti SC Cato KD Kang M-J Knaplund C. Mining clinical phrases from nursing notes to discover risk factors of patient deterioration. Int J Med Inform. (2020) 135:104053. 10.1016/j.ijmedinf.2019.104053 31884312 PMC7103062

[26] Lee E Karim H Andreescu C Mizuno A Aizenstein H Lee H. Network modeling of anxiety and psychological characteristics on suicidal behavior: Cross-sectional study. J Affect Disord. (2022) 299:545–52. 10.1016/j.jad.2021.12.050 34952111

[27] Hripcsak G Duke JD Shah NH Reich CG Huser V Schuemie MJ. Observational health data sciences and informatics (OHDSI): opportunities for observational researchers. Stud Health Technol Inform. (2015) 216:574–8. 26262116 PMC4815923

[28] Moncrieff J Crellin NE Long MA Cooper RE Stockmann T. Definitions of relapse in trials comparing antipsychotic maintenance with discontinuation or reduction for schizophrenia spectrum disorders: a systematic review. Schizophr Res. (2020) 225:47–54. 10.1016/j.schres.2019.08.035 31604607

[29] Reps JM Schuemie MJ Suchard MA Ryan PB Rijnbeek PR. Design and implementation of a standardized framework to generate and evaluate patient-level prediction models using observational healthcare data. J Am Med Inform Assoc. (2018) 25:969–75. 10.1093/jamia/ocy032 29718407 PMC6077830

[30] Jacquemin C Tzoukermann E. NLP for term variant extraction: synergy between morphology, lexicon, and syntax. In: Natural Language Information Retrieval. Dordrecht: Springer (1999). p. 25–74. 10.1007/978-94-017-2388-6_2

[31] Blei DM. Probabilistic topic models. Commun ACM. (2012) 55:77–84. 10.1145/2133806.2133826

[32] Cao J Xia T Li J Zhang Y Tang S. A density-based method for adaptive LDA model selection. Neurocomputing. (2009) 72:1775–81. 10.1016/j.neucom.2008.06.011

[33] Sommeria-Klein G Zinger L Coissac E Iribar A Schimann H Taberlet P. Latent dirichlet allocation reveals spatial and taxonomic structure in a DNA-based census of soil biodiversity from a tropical forest. Mol Ecol Resour. (2020) 20:371–86. 10.1111/1755-0998.13109 31650682

[34] Fluss R Faraggi D Reiser B. Estimation of the youden index and its associated cutoff point. Biom J. (2005) 47:458–72. 10.1002/bimj.200410135 16161804

[35] Park J You SC Jeong E Weng C Park D Roh J. A framework (SOCRATex) for hierarchical annotation of unstructured electronic health records and integration into a standardized medical database: development and usability study. JMIR Med Inform. (2021) 9:e23983. 10.2196/23983 33783361 PMC8044740

[36] Zoghbi S Vulić I Moens M-F. Latent Dirichlet allocation for linking user-generated content and e-commerce data. Inf Sci. (2016) 367:573–99. 10.1016/j.ins.2016.05.047

[37] Wang W Kiik M Peek N Curcin V Marshall IJ Rudd AG. A systematic review of machine learning models for predicting outcomes of stroke with structured data. PLoS ONE. (2020) 15:e0234722. 10.1371/journal.pone.0234722 32530947 PMC7292406

[38] Hart KL Pellegrini AM Forester BP Berretta S Murphy SN Perlis RH. Distribution of agitation and related symptoms among hospitalized patients using a scalable natural language processing method. Gen Hosp Psychiatry. (2021) 68:46–51. 10.1016/j.genhosppsych.2020.11.003 33310013 PMC7855889

[39] Boag W Kovaleva O McCoy TH Rumshisky A Szolovits P Perlis RH. Hard for humans, hard for machines: predicting readmission after psychiatric hospitalization using narrative notes. Transl Psychiatry. (2021) 11:1–6. 10.1038/s41398-020-01104-w 33431794 PMC7801508

[40] Rumshisky A Ghassemi M Naumann T Szolovits P Castro V McCoy T. Predicting early psychiatric readmission with natural language processing of narrative discharge summaries. Transl Psychiatry. (2016) 6:e921. 10.1038/tp.2015.182 27754482 PMC5315537

[41] Hajihashemi Z Popescu M. An early illness recognition framework using a temporal Smith Waterman algorithm and NLP. In: AMIA Annual Symposium Proceedings. Washington, DC: American Medical Informatics Association (2013). PMC3900198 24551357

[42] Marafino BJ Park M Davies JM Thombley R Luft HS Sing DC. Validation of prediction models for critical care outcomes using natural language processing of electronic health record data. JAMA Netw Open. (2018) 1:e185097. 10.1001/jamanetworkopen.2018.5097 30646310 PMC6324323

[43] Liu J Capurro D Nguyen A Verspoor K. Early prediction of diagnostic-related groups and estimation of hospital cost by processing clinical notes. NPJ Digital Medicine. (2021) 4:1–8. 10.1038/s41746-021-00474-9 34211109 PMC8249417

[44] Christodoulou E Ma J Collins GS Steyerberg EW Verbakel JY Van Calster B. systematic review shows no performance benefit of machine learning over logistic regression for clinical prediction models. J Clin Epidemiol. (2019) 110:12–22. 10.1016/j.jclinepi.2019.02.004 30763612

[45] Sullivan S Northstone K Gadd C Walker J Margelyte R Richards A. Models to predict relapse in psychosis: a systematic review. PLoS ONE. (2017) 12:e0183998. 10.1371/journal.pone.0183998 28934214 PMC5608199

[46] Hui CL Tang JY Leung C-M Wong GH Chang W-C Chan SK. A 3-year retrospective cohort study of predictors of relapse in first-episode psychosis in Hong Kong. Aust N Z J Psychiatry. (2013) 47:746–53. 10.1177/0004867413487229 23612934

[47] Puntis S Oke J Lennox B. Discharge pathways and relapse following treatment from early intervention in psychosis services. BJP Sych Open. (2018) 4:368–74. 10.1192/bjo.2018.50 30202598 PMC6127960

[48] Tou H Yao L Wei Z Zhuang X Zhang B. Automatic infection detection based on electronic medical records. BMC Bioinform. (2018) 19:55–63. 10.1186/s12859-018-2101-x 29671399 PMC5907141

[49] Lee SH Yu S. Changes in nursing professions' scope of practice: a pilot study using electronic nursing records. Health Policy Technol. (2018) 7:15–22. 10.1016/j.hlpt.2017.12.003

[50] Lee TY Hwang WJ Kim NS Park I Lho SK Moon S-Y. Prediction of psychosis: model development and internal validation of a personalized risk calculator. Psychol Med. (2020) 1−9. 10.1017/S0033291720004675 33315005 PMC9647536

[51] Liu R Yue Y Jiang H Lu J Wu A Geng D. A risk prediction model for post-stroke depression in Chinese stroke survivors based on clinical and socio-psychological features. Oncotarget. (2017) 8:62891. 10.18632/oncotarget.16907 28968957 PMC5609889

[52] Díaz-Caneja CM Pina-Camacho L Rodríguez-Quiroga A Fraguas D Parellada M Arango C. Predictors of outcome in early-onset psychosis: a systematic review. NPJ Schizophrenia. (2015) 1:1–10. 10.1038/npjschz.2014.5 27336027 PMC4849440

[53] Hartley S Barrowclough C Haddock G. Anxiety and depression in psychosis: a systematic review of associations with positive psychotic symptoms. Acta Psychiatr Scand. (2013) 128:327–46. 10.1111/acps.12080 23379898

[54] Pelayo-Terán JM Galán VGG Martínez-García O Tabarés-Seisdedos R Crespo-Facorro B Ayesa-Arriola R. Rates and predictors of relapse in first-episode non-affective psychosis: a 3-year longitudinal study in a specialized intervention program (PAFIP). Eur Arch Psychiatry Clin Neurosci. (2017) 267:315–23. 10.1007/s00406-016-0740-3 27796500

[55] Fusar-Poli P McGorry PD Kane JM. Improving outcomes of first-episode psychosis: an overview. World psychiatry. (2017) 16:251–65. 10.1002/wps.20446 28941089 PMC5608829

[56] Zhang Y Li H-J Wang J Cohen T Roberts K Xu H. Adapting word embeddings from multiple domains to symptom recognition from psychiatric notes. AMIA Summits Transl Sci Proc. (2018) 2017:281–9. 29888086 PMC5961810

[57] Werbeloff N Osborn DP Patel R Taylor M Stewart R Broadbent M. The Camden & islington research database: using electronic mental health records for research. PLoS ONE. (2018) 13:e0190703. 10.1371/journal.pone.0190703 29377897 PMC5788349

[58] de Jong Y Ramspek CL van der Endt VH Rookmaaker MB Blankestijn PJ Vernooij RW. A systematic review and external validation of stroke prediction models demonstrates poor performance in dialysis patients. J Clin Epidemiol. (2020) 123:69–79. 10.1016/j.jclinepi.2020.03.015 32240769

[59] Reps J Kim C Williams R Markus A Yang C Salles TD. Implementation of the COVID-19 vulnerability index across an international network of health care data sets: collaborative external validation study. JMIR Med Inform. (2021) 9:e21547. 10.2196/21547 33661754 PMC8023380

[60] Azad TD Ehresman J Ahmed AK Staartjes VE Lubelski D Stienen MN. Fostering reproducibility and generalizability in machine learning for clinical prediction modeling in spine surgery. Spine J. (2021) 21:1610–6. 10.1016/j.spinee.2020.10.006 33065274

[61] Lecomte T Giguère C-É Cloutier B Potvin S Consortium S. Comorbidity profiles of psychotic patients in emergency psychiatry. J Dual Diagn. (2020) 16:260–70. 10.1080/15504263.2020.1713425 31983294

[62] Appelbaum PS. Privacy in psychiatric treatment: threats and responses. Am J Psychiatry. (2002) 159:1809–18. 10.1176/appi.ajp.159.11.1809 12411211

[63] Chung W Cho WH Yoon CW. The influence of institutional characteristics on length of stay for psychiatric patients: a national database study in South Korea. Soc Sci Med. (2009) 68:1137–44. 10.1016/j.socscimed.2008.12.045 19167140

[64] Rasmy L Wu Y Wang N Geng X Zheng WJ Wang F. A study of generalizability of recurrent neural network-based predictive models for heart failure onset risk using a large and heterogeneous EHR data set. J Biomed Inform. (2018) 84:11–6. 10.1016/j.jbi.2018.06.011 29908902 PMC6076336

[65] Pierre JM. Diagnostic uncertainty, antipsychotic dosing, and optimal psychosocial interventions: Unanswered questions in first-episode psychosis. Schizophr Res. (2021) 228:600–1. 10.1016/j.schres.2020.11.042 33257137

[66] Baryshnikov I Sund R Marttunen M Svirskis T Partonen T Pirkola S. Diagnostic conversion from unipolar depression to bipolar disorder, schizophrenia, or schizoaffective disorder: a nationwide prospective 15-year register study on 43 495 inpatients. Bipolar Disord. (2020) 22:582–92. 10.1111/bdi.12929 32385906

[67] Francey SM O'Donoghue B Nelson B Graham J Baldwin L Yuen HP. Psychosocial intervention with or without antipsychotic medication for first-episode psychosis: a randomized noninferiority clinical trial. Schizophrenia Bulletin Open. (2020) 1:sgaa015. 10.1093/schizbullopen/sgaa015

[68] Leighton SP Krishnadas R Chung K Blair A Brown S Clark S et al. Predicting one-year outcome in first episode psychosis using machine learning. PLoS ONE. (2019) 14:e0212846. 10.1371/journal.pone.0212846 30845268 PMC6405084

