# Supplementary material for: Psychosis Relapse Prediction Leveraging Electronic Health Records Data and Natural Language Processing Enrichment Methods
Source: Front Psychiatry. 2022 Apr 5;13:844442. doi: 10.3389/fpsyt.2022.844442 (PMC9037331; doi:10.3389/fpsyt.2022.844442)
Supplement: Supplementary file 1 [file Data_Sheet_1.pdf]

Supplementary Material

Supplementary Material Appendix 1: Cohort definition

1. Patients with psychotic disorder

Initial Event Cohort

People having any of the following:

- condition occurrence of '**psychotic disorder**'

Limit initial events to: earliest event per person.

Restrict initial events to

Having any of the following criteria:

Inclusion Criteria #1: with history of antipsychotics medication

- With at least 2 occurrences of a drug exposure of ‘**antipsychotics’**, starting between 0 days before and all days after cohort entry start date.

Inclusion Criteria #2: with history of psychiatric procedure

- With at least 2 occurrences of a procedure occurrence of ‘**psychiatric procedure**’, starting between 0 days before and all days after cohort entry start date.

Limit qualifying cohort to earliest event per person.

End Date Strategy

No end date strategy selected. By default, the cohort end date will be the end of the observation period that contains the index event.

Cohort Collapse Strategy:

Collapse cohort by era with a gap size of 0 days.

2. Relapse in psychosis

Initial Event Cohort

People having any of the following:

- condition occurrences of '**psychotic disorder**' with a visit occurrence of ‘**Emergency room visit**’, ‘**Inpatient Visit**’, ‘**Emergency room and Inpatient visit**’, ‘**Ambulatory surgical center**’, ‘**Ambulatory corporate health clinic/center**’

with continuous observation of at least 0 days prior and 0 days after event index date and limit initial events to: all events per person.

Limit qualifying cohort to all events per person.

End Date Strategy

No end date strategy selected. By default, the cohort end date will be the end of the observation period that contains the index event.

Cohort Collapse Strategy:

Collapse cohort by era with a gap size of 0 days.

Supplementary Material Appendix 2: Code list for definitions

1. **Psychotic disorder**

OMOP Concept Id	OMOP Concept Name	Domain	Vocabulary	Excluded	Descendants	Mapped
436073	Psychotic disorder	Condition	SNOMED	NO	YES	NO

2. **Antipsychotics**

OMOP Concept Id	OMOP Concept Name	Domain	Vocabulary	Excluded	Descendants	Mapped
21604490	Antipsychotics	Drug	ATC	NO	YES	NO

3. **Psychiatric procedure**

OMOP Concept Id	OMOP Concept Name	Domain	Vocabulary	Excluded	Descendants	Mapped
45887951	Psychotherapy Services and Procedures	Procedure	CPT4	NO	YES	NO
4327941	Psychotherapy	Procedure	SNOMED	NO	YES	NO
2795675	Mental Health, Individual Psychotherapy	Procedure	ICD10PCS	NO	YES	NO
2795842	Mental Health, Electroconvulsive Therapy	Procedure	ICD10PCS	NO	YES	NO

4030840	Electroconvulsive therapy	Procedure	SNOMED	NO	YES	NO

4. Visit

OMOP Concept Id	OMOP Concept Name	Domain	Vocabulary	Excluded	Descendants	Mapped
262	Emergency Room and Inpatient Visit	Visit	Visit	NO	YES	NO
8883	Ambulatory Surgical Center	Visit	Visit	NO	YES	NO
9201	Inpatient Visit	Visit	Visit	NO	YES	NO
9203	Emergency Room Visit	Visit	Visit	NO	YES	NO
38004217	Ambulatory Corporate Health Clinic/Center	Visit	Visit	NO	YES	NO

Supplementary Table 1. List of the features and its variable importance given by each LASSO logistic regression model

Model 1 (Structured data only)		Model 2 (Psychological test)		Model 3 (Admission note)		Model 4 (Initial nursing assessment)		Model 5 (All note types)	
Features	Coefficient	Features	Coefficient	Features	Coefficient	Features	Coefficient	Features	Coefficient
(Intercept)	1.073	(Intercept)	0.924	(Intercept)	1.882	(Intercept)	2.077	(Intercept)	-1.290
Antipsychotics	-1.661	Topic 3	-2.602	Antipsychotics	-2.324	Antipsychotics	-2.125	Topic 1 (Psychological test)	3.254
Depressive disorder	-1.363	Topic 4	2.326	Non-drinker	-1.505	Depressive disorder	-1.714	Topic 1 (Admission note)	2.327
Non-drinker	-1.249	Topic 2	-2.310	Depressive disorder	-1.395	Non-drinker	-1.660	Non-smoker	-1.706
Non-smoker	-1.118	Depressive disorder	-1.676	Topic 1	-1.198	Non-smoker	-1.429	Non-drinker	-1.698
Male	-1.046	Antipsychotics	-1.338	Non-smoker	-1.142	Male	-0.783	Depressive disorder	-1.697
Individual psychotherapy	-0.372	Non-drinker	-1.140	Male	-0.493	Topic 2	-0.395	Antipsychotics	-1.377
		Non-smoker	-1.139	Individual psychotherapy	-0.113	Topic 3	0.101	Topic 4 (Psychological test)	1.148
		Male	-0.658			Individual psychotherapy	-0.088	Topic 1 (Initial nursing assessment)	1.000
		Topic 1	0.629			Topic 1	0.032	Male	-0.075

	Individual psychotherapy	-0.446		Topic 2 (Initial nursing assessment)	0.073
	Topic 5	0.098			

Supplementary Table 2. Performance of external validation using the MJH database

Model	N (events, %)	ACC	F1 score	AUPRC	AUROC
Model1 (structured only)	4391 (202, 4.6)	0.114	0.089	0.042	0.468
Model 4 (EMR + initial nursing assessment)	541 (33, 6.1)	0.832	0.209	0.097	0.616

N, number; ACC, accuracy; AUPRC, area under the precision-recall curve; AUROC, area under the receiver operating characteristics curve; EMR, electronic medical records

Supplementary Table 3. Baseline characteristics for the population in the MJH database

Variable	Non-relapse (n = 4,189)	Relapse (n = 202)	$\chi^2_{(df)}$	P-value
Age group, n (%)				
< 20	163 (3.9)	17 (8.4)	8.92(1)	< 0.01*
20 – 29	335 (8.0)	48 (23.8)	58.19(1)	< 0.01*
30 – 39	374 (8.9)	35 (17.3)	15.11(1)	< 0.01*
≥ 40	3,317 (79.2)	102 (50.5)	90.36(1)	< 0.01*
Sex, n (%)				
Male	1,883 (45.0)	97 (48.0)	0.61(1)	0.43
Medical history, n (%)				
Diabetes mellitus	526 (12.6)	9 (4.4)	11.08(1)	< 0.01*
Heart disease	157 (3.7)	7 (3.5)	0.01(1)	0.99
Hypertension	1,508 (36.0)	28 (13.9)	40.56(1)	< 0.01*
Psychiatric history, n (%)				
Acute transient psychotic disorder	100 (2.4)	9 (4.5)	2.60(1)	0.11
Anxiety disorder	353 (8.4)	15 (7.4)	0.14(1)	0.71
Delusional disorder	59 (1.4)	6 (3.0)	2.24(1)	0.13
Insomnia	635 (15.2)	19 (9.4)	4.59(1)	0.03*
Mood disorder	1,378 (32.9)	62 (30.7)	0.33(1)	0.57
Neurodevelopmental disorder	448 (10.7)	56 (27.7)	53.33(1)	< 0.01*
Schizoaffective disorder	313 (7.5)	20 (10.0)	1.29(1)	0.26
Schizophrenia	1,585 (37.8)	66 (32.7)	1.98(1)	0.16
Psychiatry medication use, n (%)				
Anticholinergics	232 (5.5)	23 (11.4)	11.00(1)	< 0.01*

Antidepressants	648 (15.5)	30 (14.9)	0.02(1)	0.89
Antiepileptics	209 (5.0)	11 (5.4)	0.02(1)	0.90
Antipsychotics	1,705 (40.7)	100 (49.5)	5.81(1)	0.02*
Benzodiazepine	1,665 (39.7)	97 (48.0)	5.15(1)	0.02*
Beta blocking agents	276 (6.6)	13 (6.4)	0.01(1)	1.00
Opioids	248 (5.9)	6 (3.0)	2.56(1)	0.11

Notes: $\chi^2_{(df)}$: chi square value and degree of freedom; ***** indicates statistical significance (P-value < 0.05).

Supplementary Table 4. A detailed description of the LDA for NLP applications

**Description**	Application to cluster documents of patients with psychotic disorder into topics	
**Definition**	Development approach	Machine-learning<br><br>(un-supervised clustering)
	Classes produced	If we set the number of topics, the LDA model classifies topics according to those numbers
	Detailed method	In LDA, documents are viewed as a distribution over topics while each topic is a distribution over words. LDA firstly samples a document-specific multinomial distribution over topics from a Dirichlet distribution. Then it repeatedly samples the words from these topics.[1]
**Performance**	To find the best number of topics, we used the perplexity score in this study. Perplexity scores is a statistical measure for probabilistic models, and is used to calculate the optimal number of topics in LDA. Griffiths2004 and Deveaud2014 represent maximization approaches which means that a higher numerical fit score implies a better fit. CaoJuan2009 and Arun 2010 represent minimization approaches which means that a lower numerical fit score implies a better fit. Using the perplexity score, we selected 10 as the number of topics. (Supplementary Figure 1)	

Supplementary Table 5. The month-range of variability of relapse

	**AUMC database**	**MJH database**
**Median (IQR)**	10 (7)	34 (123)
**Duration to relapse, n (%)**		
**< 3 months**	52 (83.8)	139 (68.8)
**3-6 months**	4 (6.5)	26 (12.9)
**6-9 months**	3 (4.8)	24 (11.9)
**9-12 months**	3 (4.8)	14 (6.9)

Notes: AUMC, Ajou University Medical Center; MJH, Myongji Hospital; IQR, interquartile

Supplementary Figure 1. The perplexity graphs which indicate the optimal number of LDA topics

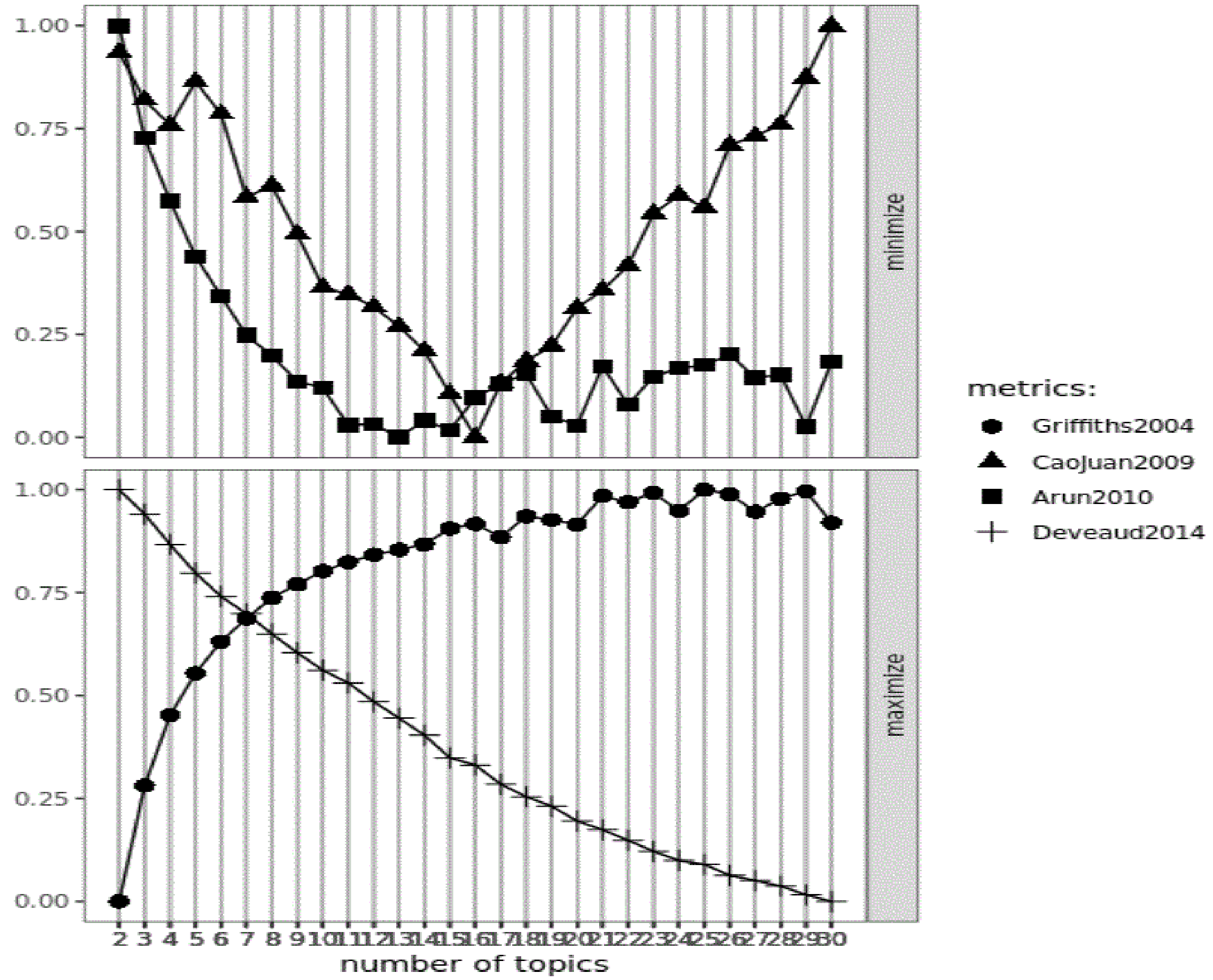

Reference

1. Cao J, Xia T, Li J, Zhang Y, Tang S. A density-based method for adaptive LDA model selection. *Neurocomputing*. 2009;72(7-9):1775-1781.
